# Supplementary material for: DAF-16/FOXO and HLH-30/TFEB function as combinatorial transcription factors to promote stress resistance and longevity
Source: Nat Commun. 2018 Oct 23;9:4400. doi: 10.1038/s41467-018-06624-0 (PMC6199276; doi:10.1038/s41467-018-06624-0)
Supplement: Supplementary file 1 — Supplementary Information [file 41467_2018_6624_MOESM1_ESM.pdf]

Lin et al., Supplementary Figure 1

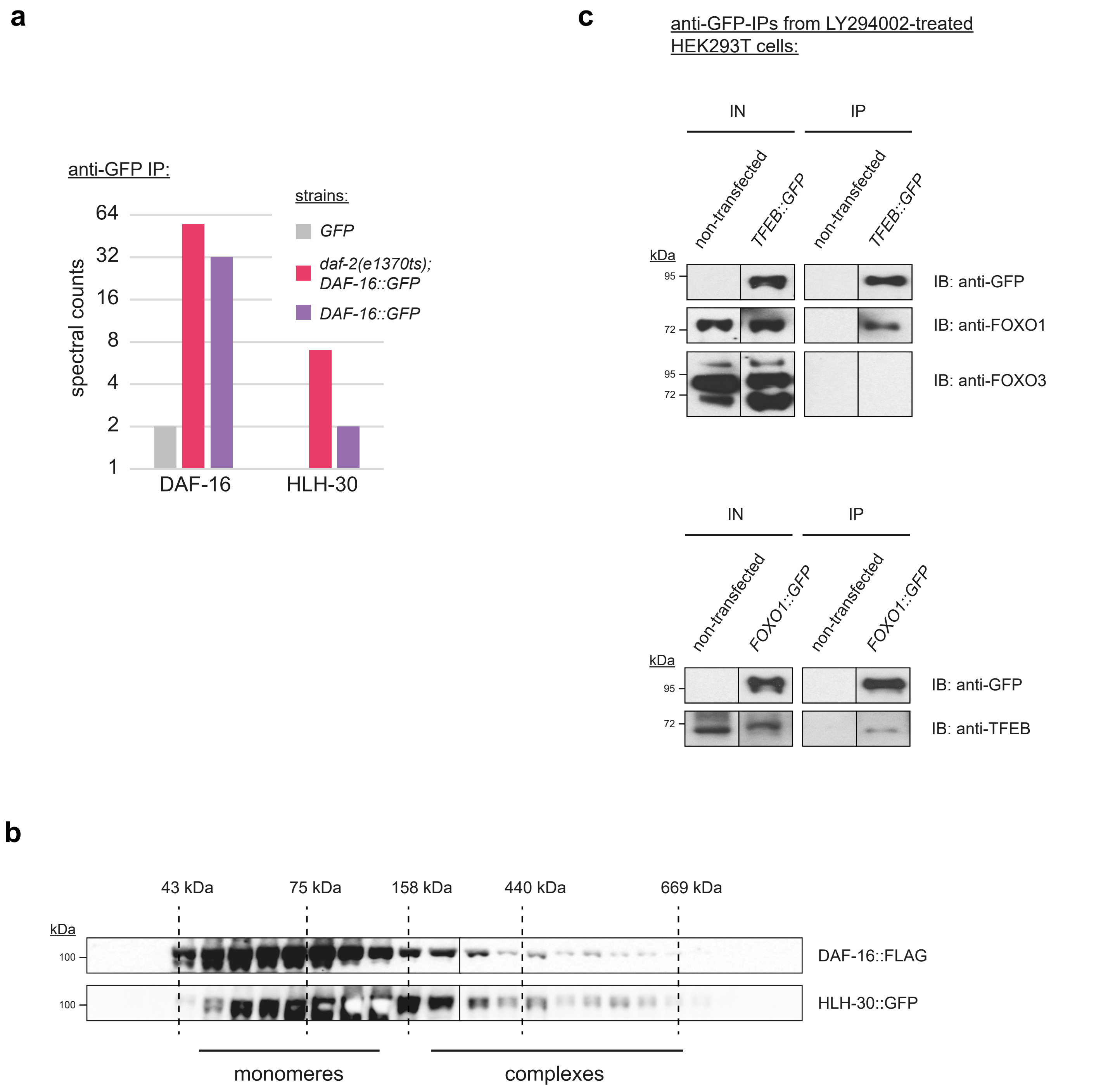

Supplementary Figure 1 - Confirmatory IPs from *C. elegans* and human HEK293T cells as well as size-exclusion chromatography illustrating the size distribution of DAF-16 and HLH-30 containing complexes in vivo.

a Large-scale immunoprecipitations conducted in identical manner to Figure 1a and 1b, but using a different DAF-16::GFP transgene and a different anti-GFP antibody. **b** Full weight-spectrum of the size-exclusion chromatography from Figure 1e, illustrating the size distributions of DAF-16 and HLH-30 in wild type/untreated *C. elegans*. Elution fractions were analyzed by SDS-PAGE and western blotting. Weight ranges in which monomeric or complex-incorporated forms of DAF-16 and HLH-30 are expected to run are indicated. **c** co-IPs of human TFEB, FOXO1, and FOXO3 in HEK293T cells. HEK293T cells expressing either TFEB::GFP, FOXO1::GFP, or non-tranfected controls were treated with the PI3 kinase inhibitor LY-294002, lysed and the tagged proteins immunoprecipitated using GFP-Trap resin. Samples were analyzed by SDS-PAGE and western blotting. Inputs (IN) and eluates (IP) of the co-IPs were analyzed by SDS-PAGE and western blotting. For the inputs (IN), only fractions were loaded: 50% for the anti-GFP western blots and 10% for the anti-FOXO1, anti-FOXO3, and anti-TFEB western blots. IB: antibody used for immunoblot.

Lin et al., Supplementary Figure 2

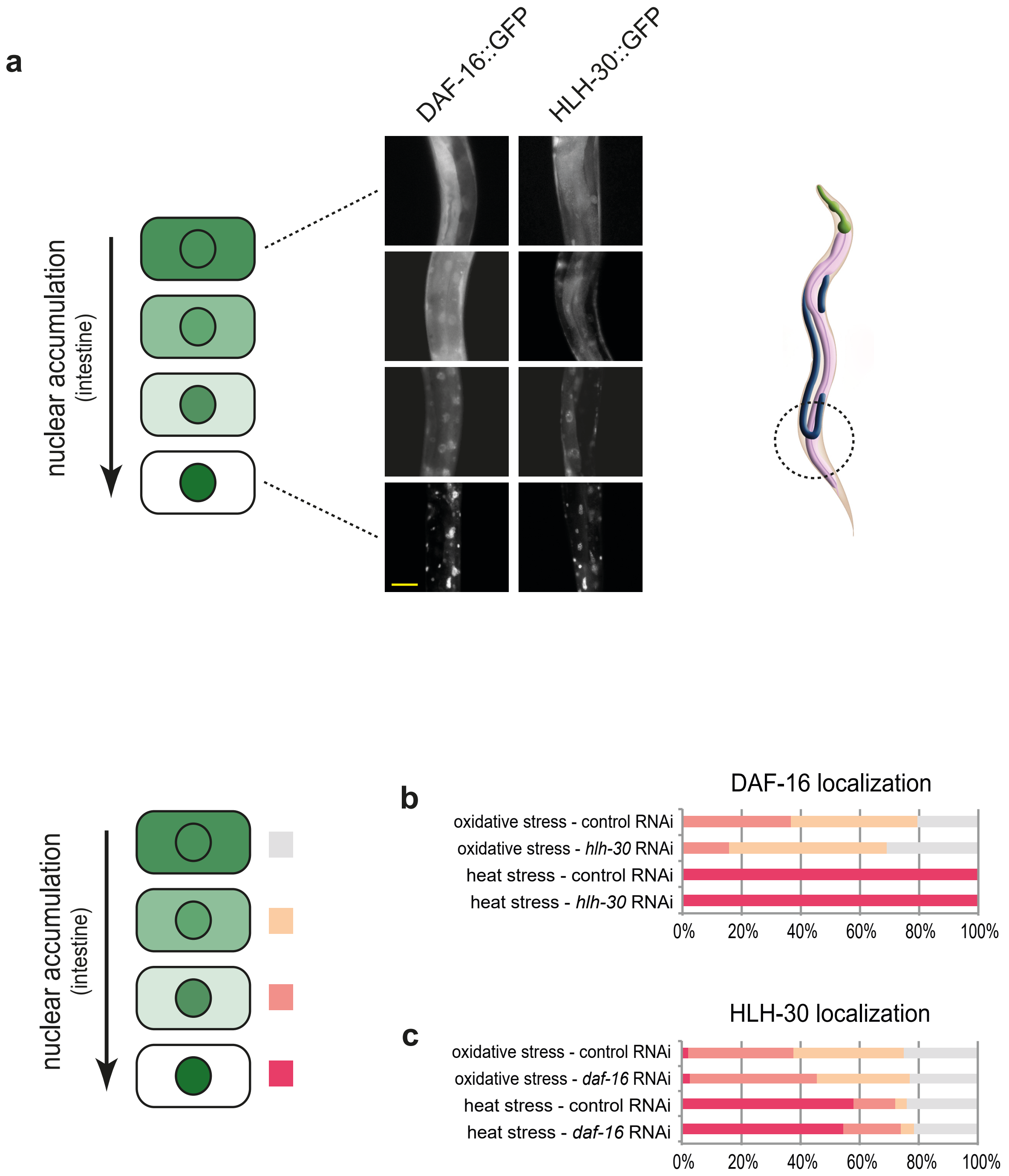

Supplementary Figure 2 - DAF-16 and HLH-30 do not depend on each other for their nuclear translocation.

a Representative images of the different categories of nuclear accumulation scored in Figures 2c and Supplementary Figure 2b,c. Yellow scale bar: 40 µm. (The anatomical sketch was adapted from wormatlas.org.) **b,c** DAF-16::GFP (**b**) or HLH-30::GFP (**c**) expressing animals were grown from L1 on the indicated RNAi bacteria. Nuclear translocation of the GFP signal was scored in day 2 adult animals, either upon oxidative stress (12 h on 100 mM tBOOH) or heat stress (1 h at 35°C). (n=100)

Lin et al., Supplementary Figure 3

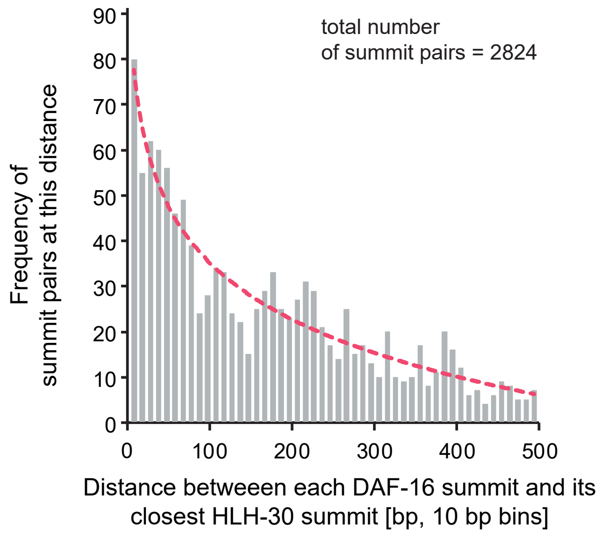

Supplementary Figure 3 - There is preferentially no spacing between the nearest DAF-16 and HLH-30 bound sites in the genome.

Spacing between the summits of each DAF-16 bound site and their closest summit of a HLH-30 bound site. A total of 2824 pairs were determined and their distances plotted in 10 bp bins. Only distances below 500 bp are shown. The red line represents a trendline.

Lin et al., Supplementary Figure 4

a

sites only bound by DAF-16	e-value	sites co-bound by DAF-16 and HLH-30 (searched window is centered on DAF-16 summits)	e-value

over-represented motifs:

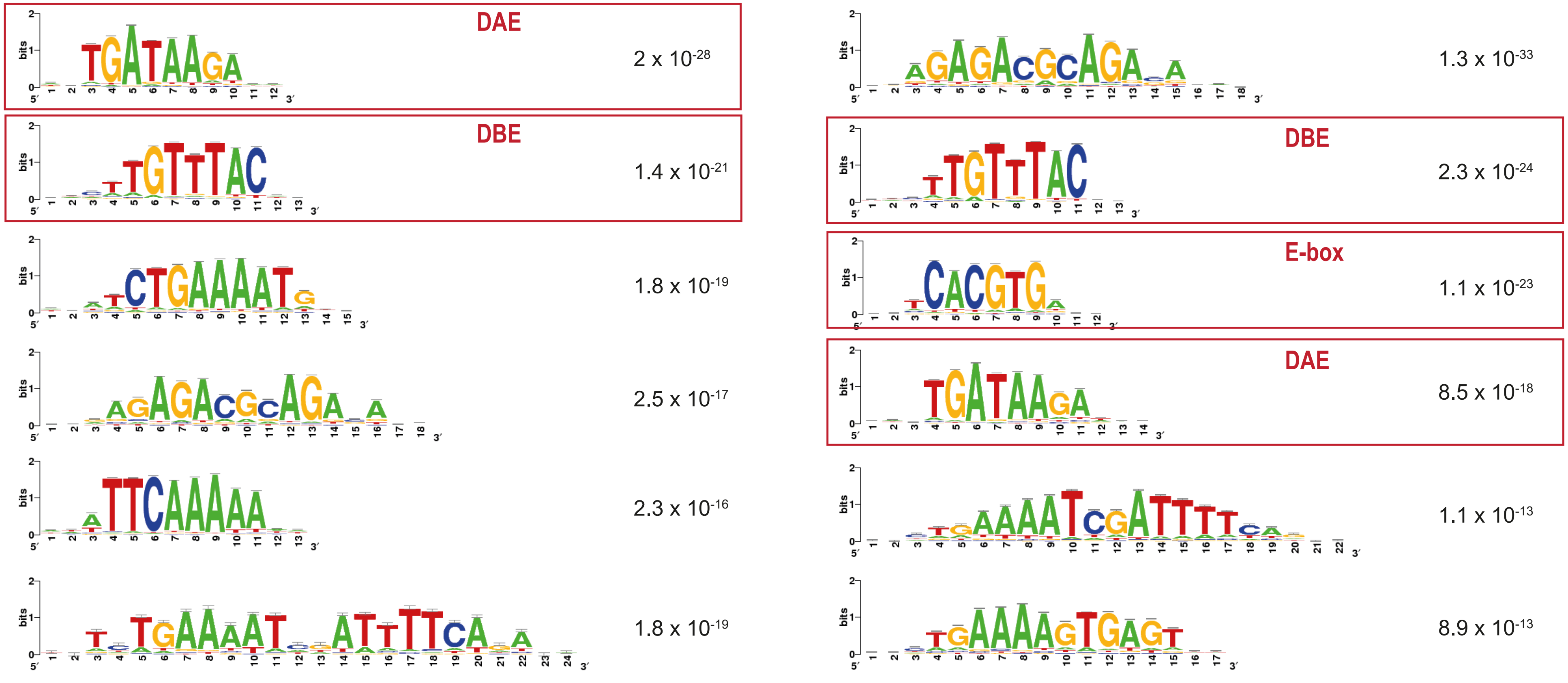

additional motifs, showing a positional bias: none

additional motifs, showing a positional bias: none

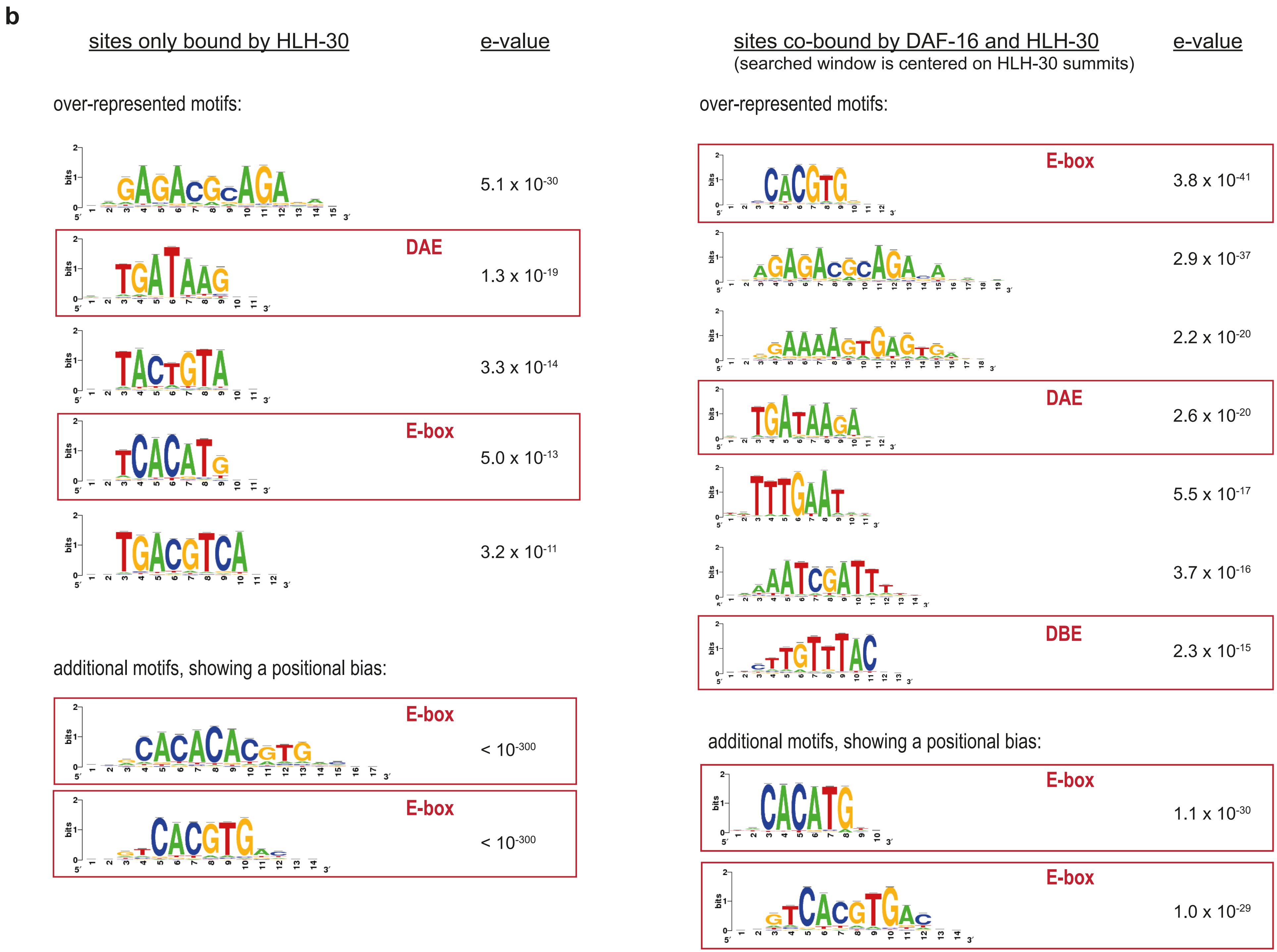
b
sites only bound by HLH-30
e-value
over-represented motifs:
5.1 x 10-30
DAE
1.3 x 10-19
3.3 x 10-14
E-box
5.0 x 10-13
3.2 x 10-11
additional motifs, showing a positional bias:
E-box
< 10-300
E-box
< 10-300
sites co-bound by DAF-16 and HLH-30
(searched window is centered on HLH-30 summits)
e-value
over-represented motifs:
E-box
3.8 x 10-41
2.9 x 10-37
2.2 x 10-20
DAE
2.6 x 10-20
5.5 x 10-17
3.7 x 10-16
DBE
2.3 x 10-15
additional motifs, showing a positional bias:
E-box
1.1 x 10-30
E-box
1.0 x 10-29

Supplementary Figure 4 - Sequence motifs enriched at sites bound only by DAF-16, only by HLH-30, or by both transcription factors.

Previous work had shown that DAF-16 bound sites are enriched for DAF-16-Bound Elements (DBEs, TRTTTAC) and DAF-16-Associated Elements (DAEs, TGATAAG)[1,2]. On the other hand, helix-loop-helix transcription factors are known to bind E-boxes (CANNTG)[3], with a recent study using nucleotide binding arrays to identify the E-box CACGTG as the preferred sequence bound by HLH-30 in vitro[4]. Consistently, ChIP-Seq studies of the mammalian HLH-30 ortholog TFEB have shown that it binds to the E-box CACGTG at the core of a so-called CLEAR motif (GTCACGTGAC)[5].

Using our own ChIP-Seq data from Figure 5, we here conducted de novo motif discovery using RSAT peak-motifs (http://metazoa.rsat.eu/)[6] on the sites bound only by DAF-16, only by HLH-30, or both transcription factors. As one might expect, the DBE motif emerged amongst the most significantly enriched motifs at sites bound only by DAF-16 or co-bound by DAF-16 and HLH-30, while it was not enriched at sites bound only by HLH-30 (**a**). Conversely, our de novo search for enriched motifs identified E-boxes at sites only bound by HLH-30 and at those co-bound by both transcription factors but not at those bound only by DAF-16 (**b**). Interestingly, DAF-16-Associated Elements (DAEs, TGATAAG) were found enriched at all three types of sites: those bound only by DAF-16, those bound only by HLH-30, and those bound by both transcription factors.

During this analysis, all e-values were generated by RSAT[6] and only motifs with e-values smaller than 10^{-10} were considered significant. DAF-16-Bound Elements (DBE), DAF-16-Associated Elements (DAE), and E-boxes are highlighted in red.

Lin et al., Supplementary Figure 5

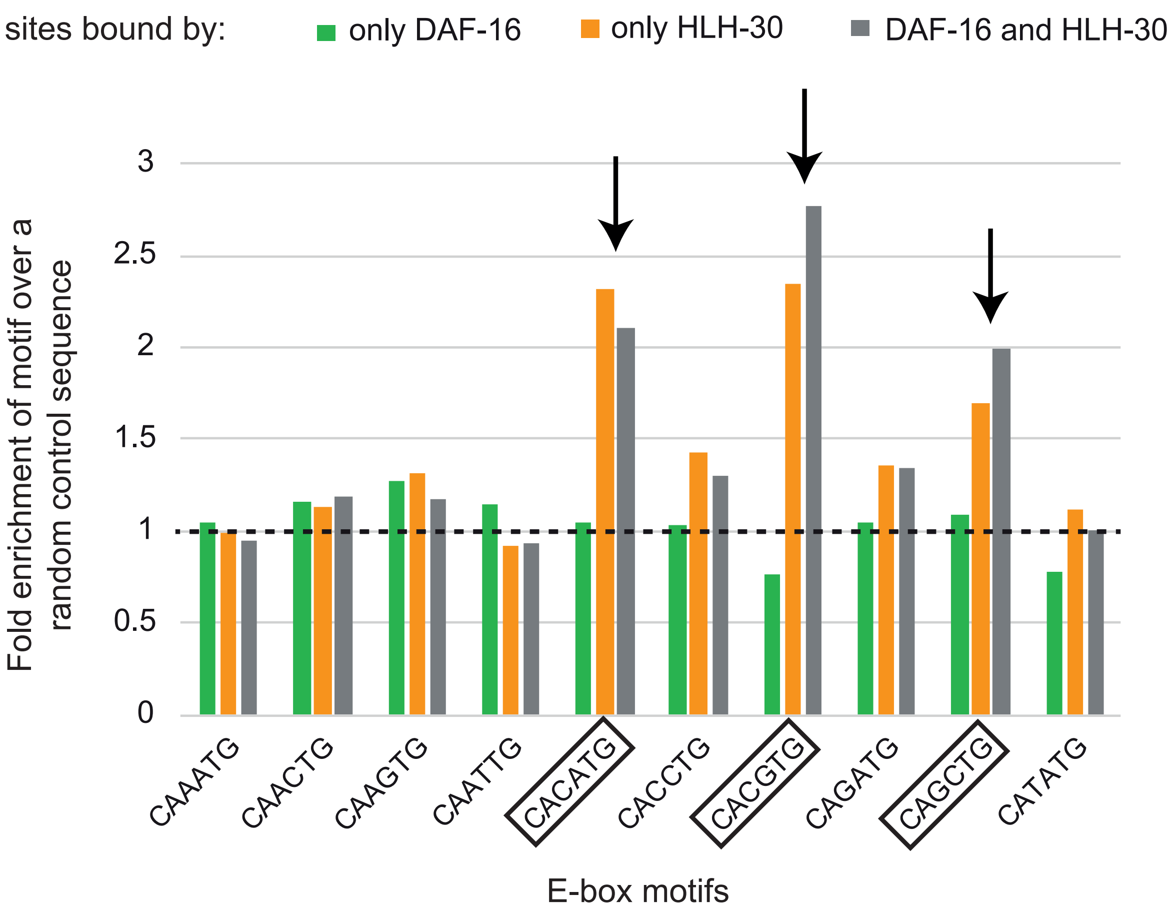

Supplementary Figure 5 - HLH-30 binds to at least three different E-boxes in vivo.

We noted that the E-boxes identified in Supplementary Figure 4 were diverse - not limited to the previously implicated CACGTG. Using again the ChIP-seq data from Figure 5, we determined the abundance of all possible E-boxes at sites bound only by DAF-16, only by HLH-30, or by both transcription factors. Enrichment of the different E-boxes over their abundance in a randomized control sequence is shown. Arrows indicate enrichments greater than 1.5-fold. Although CACGTG is the most enriched motif (~2.5-fold enriched), also other E-boxes, specifically CACATG and CAGCTG, emerged as ~2.1 and ~1.8-fold enriched, respectively, suggesting that the motif specificity of HLH-30 in vivo is broader than previously thought[4,5]. The balance between these different E-boxes did not substantially differ between sites bound only by HLH-30 and those co-bound by HLH-30 and DAF-16 and thus was likely not influenced by presence or absence of DAF-16.

Lin et al., Supplementary Figure 6

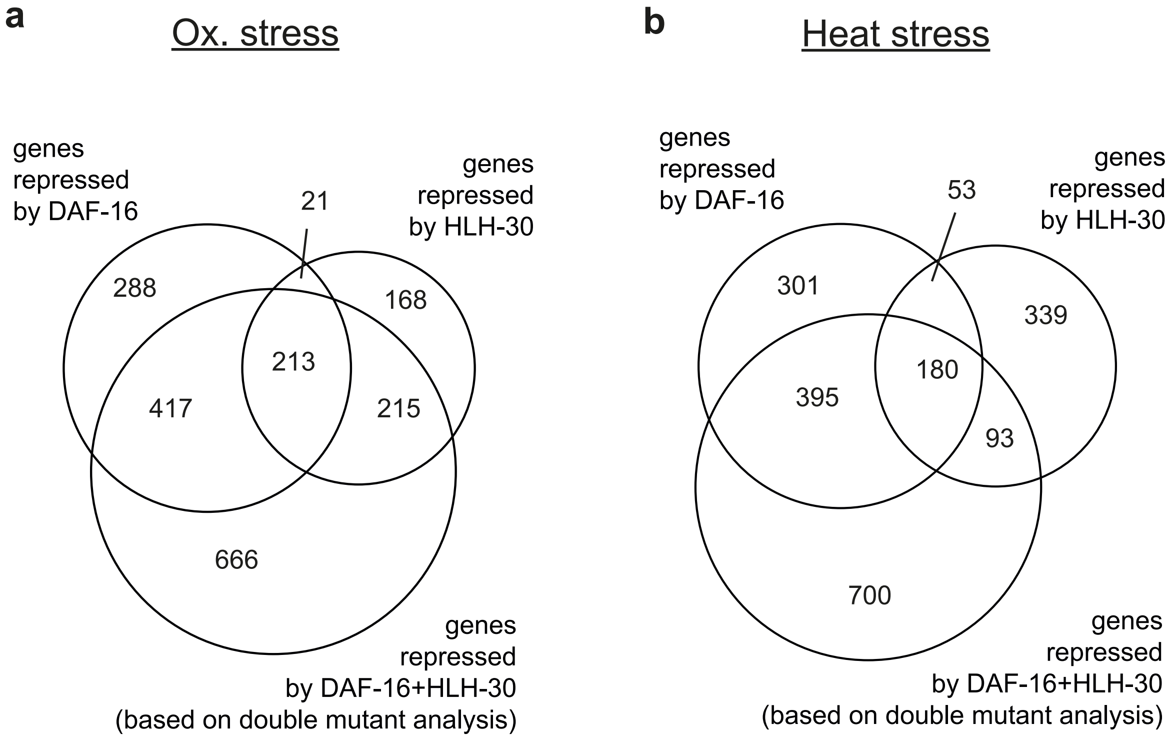

Supplementary Figure 6 - Venn diagrams illustrating that DAF-16 and HLH-30 not only synergize in gene activation but also in gene repression in response to oxidative stress or heat stress.

C. elegans with the genotypes wild type, *daf-16(mu86lf)*, *hlh-30(tm1978lf)*, and *daf-16(mu86lf)*; *hlh-30(tm1978lf)* were grown at 20°C until young adulthood, then transferred for 12 h to either 6mM tBOOH (oxidative stress) (**a**) or to 32°C (heat stress) (**b**), harvested, and their transcriptomes determined by mRNA-seq. The Venn diagrams illustrate the number of genes significantly repressed by DAF-16 and/or HLH-30, based on the comparison of their mutants to wild type animals (for Venn diagrams of the activated genes see Fig. 7c,d). (Significance of gene expression changes was determined by Cuffdiff, using an FDR of 0.05.)

Lin et al., Supplementary Figure 7

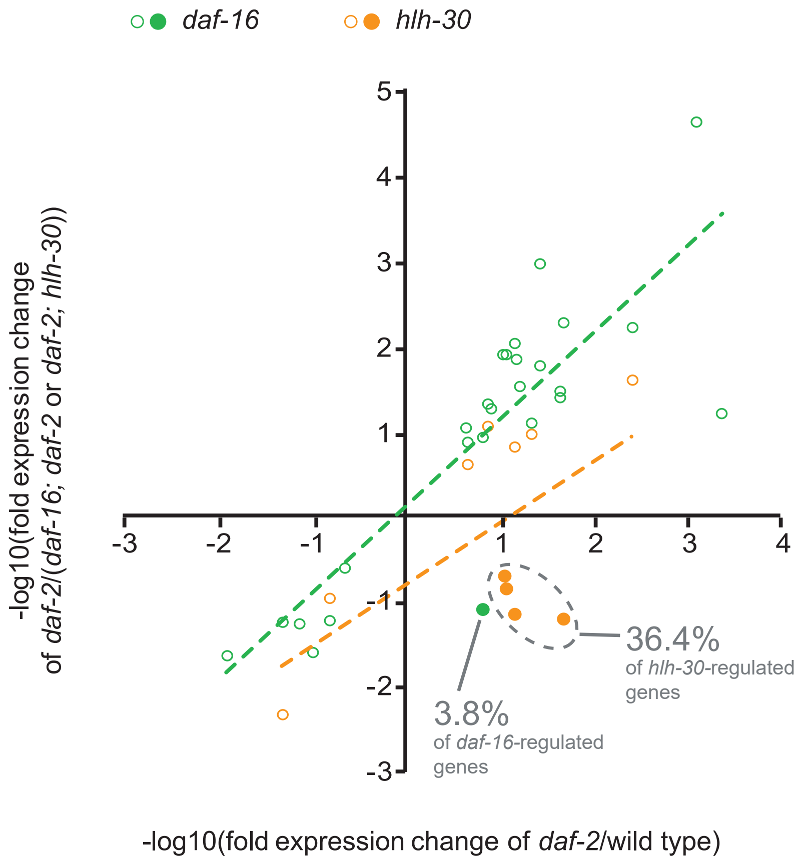

Supplementary Figure 7 - In contrast to DAF-16, HLH-30 actively opposes some of the dauer formation-related gene expression changes in *daf-2(e1370ts)* animals.

Regulation of dauer formation-related genes by *daf-2*, *daf-16*, and *hlh-30*. Scatter plot based on the gene expression data from Figure 4, illustrating the differential expression of dauer formation-related genes (n=276, see Fig. 7e)) upon inactivation of *daf-2* (x-axis) and their regulation by either *daf-16* (*daf-2(e1370ts)* over *daf-2(e1370ts)*; *daf-16(mgDf47lf)*) or *hlh-30* (*daf-2(e1370ts)* over *daf-2(e1370ts)*; *hlh-30(tm1978lf)*) (y-axis). Only genes significantly regulated in both dimensions are shown ($p_{corr}<0.05$; p_{corr} was determined using Cuffdiff (see methods)). As one would expect from a key regulator of *daf-2(e1370ts)*-mediated dauer formation, DAF-16-regulates the expression of dauer formation-related genes in a manner that highly correlates with their regulation by *daf-2(e1370ts)*. Only 3.8% of these DAF-16-regulated genes show an inverse correlation. In contrast, HLH-30 regulates 36.4% of the dauer formation-related genes in a direction that opposes their regulation in *daf-2(e1370ts)* animals. Consistently, the linear regression of the data points for HLH-30-dependent genes is shifted to the bottom right quadrant. We conclude that in contrast to DAF-16, HLH-30 actively opposes some of the gene expression changes in dauer formation-related genes that normally would occur in *daf-2(e1370ts)* animals, which we propose to be causal for the moderately enhanced dauer formation that occurs in *daf-2(e1370ts)*; *hlh-30(tm1978lf)* animals (Fig. 6c).

Lin et al., Supplementary Figure 8

a

	N2_1	N2_2	N2_3	daf16_1	daf16_2	daf16_3	hlh30_1	hlh30_2	hlh30_3
N2_1	1.000	0.882	0.930	0.876	0.859	0.906	0.975	0.924	0.979
N2_2	0.882	1.000	0.740	0.992	0.988	0.987	0.824	0.979	0.938
N2_3	0.930	0.740	1.000	0.721	0.698	0.755	0.975	0.784	0.870
daf16_1	0.876	0.992	0.721	1.000	0.996	0.993	0.813	0.981	0.938
daf16_2	0.859	0.988	0.698	0.996	1.000	0.992	0.795	0.977	0.928
daf16_3	0.906	0.987	0.755	0.993	0.992	1.000	0.845	0.992	0.961
hlh30_1	0.975	0.824	0.975	0.813	0.795	0.845	1.000	0.874	0.944
hlh30_2	0.924	0.979	0.784	0.981	0.977	0.992	0.874	1.000	0.975
hlh30_3	0.979	0.938	0.870	0.938	0.928	0.961	0.944	0.975	1.000

average correlation between replicates = 0.925

d

	N2_1	N2_2	N2_3	N2_o_1	N2_o_2	N2_o_3	daf16_o_1	daf16_o_2	daf16_o_3	hlh30_o_1	hlh30_o_2	hlh30_o_3	daf16hlh30_o_1	daf16hlh30_o_2	daf16hlh30_o_3
N2_1	1.000	0.882	0.930	0.876	0.979	0.979	0.916	0.906	0.883	0.902	0.971	0.942	0.822	0.874	0.898
N2_2	0.882	1.000	0.740	0.990	0.907	0.908	0.971	0.977	0.983	0.976	0.913	0.950	0.983	0.985	0.974
N2_3	0.930	0.740	1.000	0.735	0.872	0.876	0.765	0.752	0.724	0.753	0.857	0.804	0.663	0.713	0.740
N2_o_1	0.876	0.990	0.735	1.000	0.919	0.921	0.978	0.979	0.983	0.983	0.921	0.956	0.983	0.985	0.971
N2_o_2	0.979	0.907	0.872	0.919	1.000	0.998	0.958	0.945	0.926	0.946	0.991	0.977	0.868	0.917	0.935
N2_o_3	0.979	0.908	0.876	0.921	0.998	1.000	0.956	0.944	0.926	0.946	0.991	0.975	0.870	0.918	0.935
daf16_o_1	0.916	0.971	0.765	0.978	0.958	0.956	1.000	0.993	0.990	0.991	0.961	0.985	0.957	0.983	0.985
daf16_o_2	0.906	0.977	0.752	0.979	0.945	0.944	0.993	1.000	0.996	0.993	0.950	0.982	0.961	0.991	0.995
daf16_o_3	0.883	0.983	0.724	0.983	0.926	0.926	0.990	0.996	1.000	0.991	0.934	0.972	0.972	0.995	0.993
hlh30_o_1	0.902	0.976	0.753	0.983	0.946	0.946	0.991	0.993	0.991	1.000	0.958	0.987	0.964	0.992	0.990
hlh30_o_2	0.971	0.913	0.857	0.921	0.991	0.991	0.961	0.950	0.934	0.958	1.000	0.985	0.883	0.932	0.947
hlh30_o_3	0.942	0.950	0.804	0.956	0.977	0.975	0.985	0.982	0.972	0.987	0.985	1.000	0.928	0.969	0.980
daf16hlh30_o_1	0.822	0.983	0.663	0.983	0.868	0.870	0.957	0.961	0.972	0.964	0.883	0.928	1.000	0.984	0.963
daf16hlh30_o_2	0.874	0.985	0.713	0.985	0.917	0.918	0.983	0.991	0.995	0.992	0.932	0.969	0.984	1.000	0.993
daf16hlh30_o_3	0.898	0.974	0.740	0.971	0.935	0.935	0.985	0.995	0.993	0.990	0.947	0.980	0.963	0.993	1.000

average correlation between replicates = 0.949

b

	N2_1	N2_2	daf2_1	daf2_2	daf2daf16_1	daf2daf16_2	daf2hlh30_1	daf2hlh30_2
N2_1	1.000	0.985	0.308	0.315	0.334	0.333	0.325	0.313
N2_2	0.985	1.000	0.300	0.306	0.326	0.324	0.311	0.301
daf2_1	0.308	0.300	1.000	0.992	0.959	0.953	0.956	0.959
daf2_2	0.315	0.306	0.992	1.000	0.963	0.964	0.971	0.970
daf2daf16_1	0.334	0.326	0.959	0.963	1.000	0.994	0.952	0.949
daf2daf16_2	0.333	0.324	0.953	0.964	0.994	1.000	0.952	0.945
daf2hlh30_1	0.325	0.311	0.956	0.971	0.952	0.952	1.000	0.983
daf2hlh30_2	0.313	0.301	0.959	0.970	0.949	0.945	0.983	1.000

average correlation between replicates = 0.989

e

	N2_1	N2_2	N2_3	N2_h_1	N2_h_2	N2_h_3	daf16_h_1	daf16_h_2	daf16_h_3	hlh30_h_1	hlh30_h_2	hlh30_h_3	daf16hlh30_h_1	daf16hlh30_h_2	daf16hlh30_h_3
N2_1	1.000	0.882	0.930	0.836	0.813	0.786	0.874	0.865	0.848	0.931	0.896	0.926	0.819	0.791	0.786
N2_2	0.882	1.000	0.740	0.690	0.969	0.960	0.940	0.927	0.934	0.878	0.923	0.859	0.960	0.954	0.953
N2_3	0.930	0.740	1.000	0.860	0.652	0.631	0.710	0.700	0.680	0.817	0.749	0.818	0.650	0.617	0.611
N2_h_1	0.836	0.690	0.860	1.000	0.711	0.690	0.780	0.777	0.752	0.898	0.825	0.902	0.717	0.686	0.681
N2_h_2	0.813	0.969	0.652	0.711	1.000	0.994	0.962	0.951	0.961	0.889	0.942	0.871	0.990	0.990	0.990
N2_h_3	0.786	0.960	0.631	0.690	0.994	1.000	0.941	0.932	0.943	0.866	0.924	0.850	0.979	0.983	0.983
daf16_h_1	0.874	0.940	0.710	0.780	0.962	0.941	1.000	0.995	0.993	0.949	0.983	0.936	0.978	0.964	0.962
daf16_h_2	0.865	0.927	0.700	0.777	0.951	0.932	0.995	1.000	0.997	0.945	0.979	0.934	0.970	0.958	0.956
daf16_h_3	0.848	0.934	0.680	0.752	0.961	0.943	0.993	0.997	1.000	0.928	0.973	0.916	0.979	0.970	0.968
hlh30_h_1	0.931	0.878	0.817	0.898	0.889	0.866	0.949	0.945	0.928	1.000	0.976	0.990	0.902	0.876	0.871
hlh30_h_2	0.896	0.923	0.749	0.825	0.942	0.924	0.983	0.979	0.973	0.976	1.000	0.966	0.958	0.940	0.936
hlh30_h_3	0.926	0.859	0.818	0.902	0.871	0.850	0.936	0.934	0.916	0.990	0.966	1.000	0.885	0.861	0.856
daf16hlh30_h_1	0.819	0.960	0.650	0.717	0.990	0.979	0.978	0.970	0.979	0.902	0.958	0.885	1.000	0.997	0.996
daf16hlh30_h_2	0.791	0.954	0.617	0.686	0.990	0.983	0.964	0.958	0.970	0.876	0.940	0.861	0.997	1.000	0.999
daf16hlh30_h_3	0.786	0.953	0.611	0.681	0.990	0.983	0.962	0.956	0.968	0.871	0.936	0.856	0.996	0.999	1.000

average correlation between replicates = 0.924

c

	N2_1	N2_2	glp1_1	glp1_2	glp1daf16_1	glp1daf16_2	glp1hlh30_1	glp1hlh30_2
N2_1	1.000	0.996	0.601	0.618	0.700	0.688	0.659	0.665
N2_2	0.996	1.000	0.599	0.617	0.699	0.692	0.659	0.664
glp1_1	0.601	0.599	1.000	0.994	0.904	0.867	0.935	0.964
glp1_2	0.618	0.617	0.994	1.000	0.911	0.859	0.938	0.965
glp1daf16_1	0.700	0.699	0.904	0.911	1.000	0.838	0.981	0.969
glp1daf16_2	0.688	0.692	0.867	0.859	0.838	1.000	0.841	0.878
glp1hlh30_1	0.659	0.659	0.935	0.938	0.981	0.841	1.000	0.988
glp1hlh30_2	0.665	0.664	0.964	0.965	0.969	0.878	0.988	1.000

average correlation between replicates = 0.954

f

	Daf16_1	Daf16_2	Hlh30_1	Hlh30_2
Daf16_1	1.000	0.927	0.312	0.397
Daf16_2	0.927	1.000	0.351	0.444
Hlh30_1	0.312	0.351	1.000	0.957
Hlh30_2	0.397	0.444	0.957	1.000

average correlation between replicates = 0.942

Supplementary Figure 8 - Correlations between replicates and conditions of the mRNA-seq and ChIP-seq experiments presented in this study.

a-e Pearson correlations between replicates and conditions for the mRNA-seq data presented in Figures 4 and 7. **f** Pearson correlations between replicates and conditions for the ChIP-seq data presented in Figures 5 and Supplementary Figures 3 to 5.

Lin et al., Supplementary Figure 9

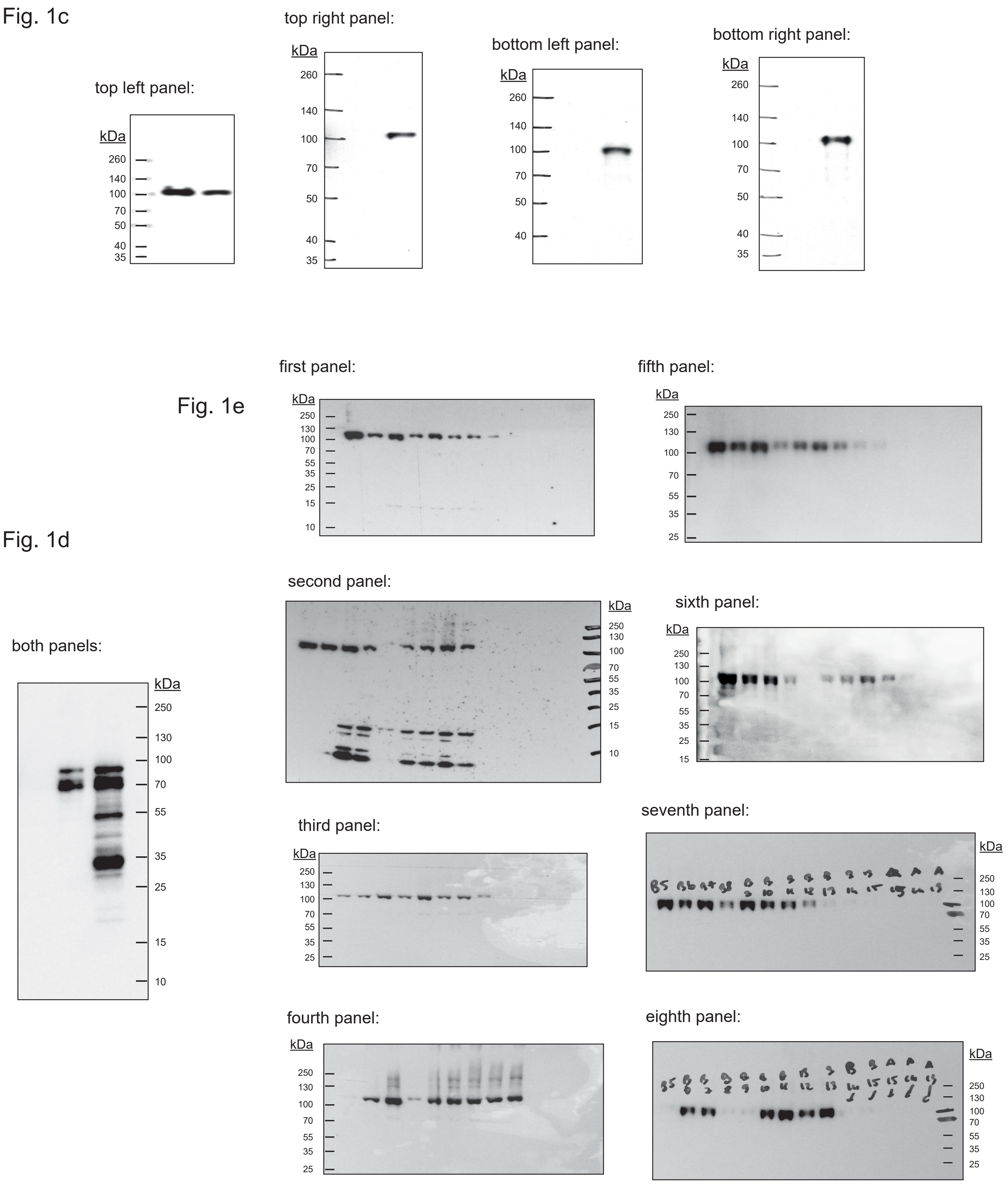

Supplementary Figure 9 - Non-cropped electrophoresis data of Figure 1.

Lin et al., Supplementary Figure 10

Suppl. Fig. 1b

top panel:

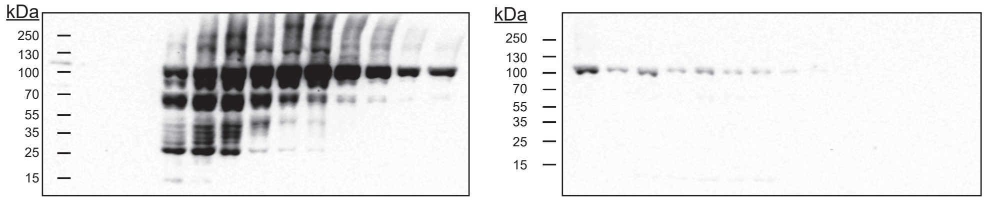

bottom panel:

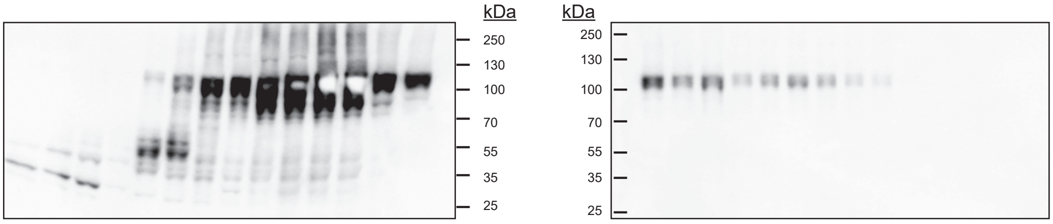

Suppl. Fig. 1c

1st row:

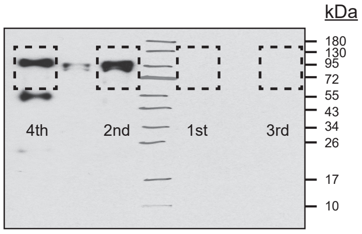

2nd row:

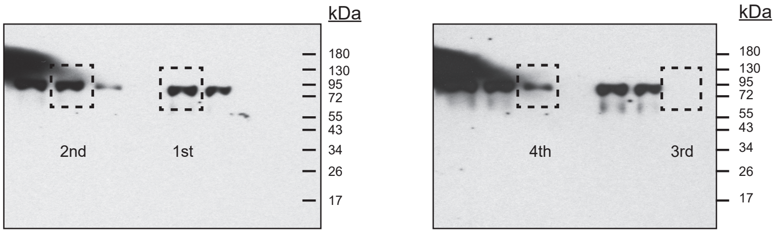

3rd row:

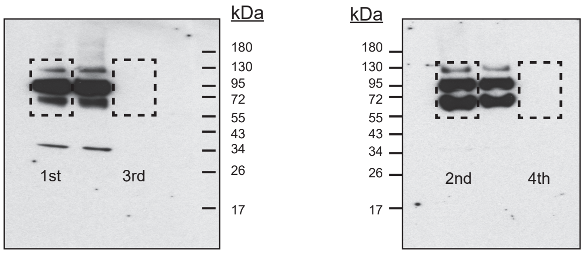

4th row:

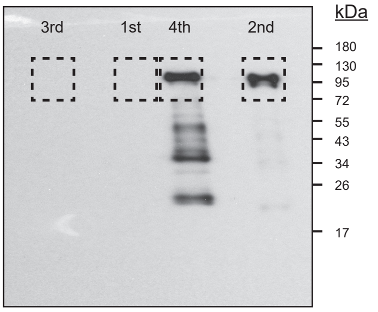

5th row:

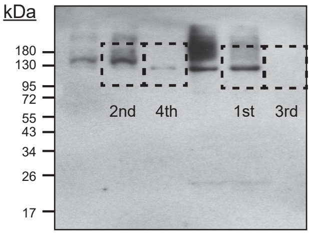

Supplementary Figure 10 - Non-cropped electrophoresis data of Supplementary Figure 1.

Lin et al., Supplementary Table 1

C. elegans strains used in this study

strain name	genotype
N2	wild type
TJ356	*zIs356[daf-16P::daf-16::GFP; rol-6(su1006)]IV*
GR1895	*daf-2(e1370ts)III; zIs356[daf-16P::daf-16::GFP; rol-6(su1006)]IV*
GR1894	*daf-18(mg198lf)IV; zIs356[daf-16P::daf-16::GFP; rol-6(su1006)]IV*
RIE470	*daf-2(e1370ts)III; Is[daf-16P::FLAG::daf-16b; rol-6(su1006)]*
RIE402	*daf-2(e1370ts)III; atrIs1[hlh-30P::hlh-30::GFP; rol-6(su1006)]; Is[daf-16P::FLAG::daf-16b; rol-6(su1006)]*
RIE529	*atrIs1[hlh-30P::hlh-30::GFP; rol-6(su1006)]; Is[daf-16P::FLAG::daf-16b; rol-6(su1006)]*
RIE330	*atrIs1[hlh-30P::hlh-30::GFP; rol-6(su1006)]*
RIE328	*daf-2(e1370ts)III; atrIs1[hlh-30P::hlh-30::GFP; rol-6(su1006)]*
CF1934	*daf-16(mu86lf)I; muIs109[daf-16P::GFP::daf-16; odr-1P::RFP]*
CF1935	*daf-16(mu86lf)I; glp-1(e2141ts)III; muIs109[daf-16P::GFP::daf-16; odr-1P::RFP]*
MAH235	*sqIs19[hlh-30P::hlhl-30::gfp; rol-6(su1006)]*
MAH262	*glp-1(e2141ts)III; sqIs19[hlh-30P::hlhl-30::gfp; rol-6(su1006)]*
CF1038	*daf-16(mu86lf)I*
RIE237	*hlh-30(tm1978lf)IV*
RIE301	*daf-16(mu86lf)I; hlh-30(tm1978lf)IV*
CB1370	*daf-2(e1370ts)III*
GR1309	*daf-16(mgDf47lf)I; daf-2(e1370ts)III*
RIE254	*daf-2(e1370ts)III; hlh-30(tm1978lf)IV*
RIE264	*daf-16(mgDf47lf)I; daf-2(e1370ts)III; hlh-30(tm1978lf)IV*
CF1903	*glp-1(e2141ts)III*
CF1880	*daf-16(mu86lf)I; glp-1(e2141ts)III*
MAH206	*glp-1(e2141ts)III; hlh-30(tm1978lf)IV*
RIE426	*daf-16(mu86lf)I; glp-1(e2141ts)III; hlh-30(tm1978lf)IV*
RIE530	*daf-16(mu86lf)I; daf-2(e1370ts)III; atrIs1[hlh-30P::hlh-30::GFP; rol-6(su1006)]*
RIE531	*daf-2(e1370ts)III; hlh-30(tm1978lf)IV; zIs356[daf-16P::daf-16::GFP; rol-6(su1006)]IV*

Lin et al., Supplementary Table 2

Summary of the survival data and their statistics

Figure 2a

	n	mean survival time [days]	Std.Error	independent replicate experiment (n; mean survival time [days])
wild type	101	14.11	0.25	270; 10.55
daf-16(mu86)	101	10.04	0.16	283; 8.40
hlh-30(tm1978)	100	8.94	0.06	255; 7.30
daf-16(mu86); hlh-30(tm1978)	98	8.33	0.13	338; 6.74

	change [%]	Std.Error	log-rank test [corr. p-value]
wild type -> daf-16(mu86)	-28.84	2.09	< 0.0000000001
wild type -> hlh-30(tm1978)	-36.64	1.81	< 0.0000000001
wild type -> daf-16(mu86); hlh-30(tm1978)	-40.98	1.98	< 0.0000000001
hlh-30(tm1978) -> daf-16(mu86); hlh-30(tm1978)	-6.86	1.57	0.0002

Figure 2b

	n	mean survival time [days]	Std.Error	independent replicate experiment (n; mean survival time [days])
daf-2(e1370)	99	34.41	0.85	149; 32.64
daf-16(mgDf47); daf-2(e1370)	106	10.39	0.17	129; 12.38
daf-2(e1370); hlh-30(tm1978)	101	15.42	0.65	152; 19.83
daf-16(mgDf47); daf-2(e1370); hlh-30(tm1978)	104	8.35	0.13	264; 9.13

	change [%]	Std.Error	log-rank test [corr. p-value]
daf-2(e1370) -> daf-16(mgDf47); daf-2(e1370)	-69.81	2.52	< 0.0000000001
daf-2(e1370) -> daf-2(e1370); hlh-30(tm1978)	-55.19	3.11	< 0.0000000001
daf-2(e1370) -> daf-16(mgDf47); daf-2(e1370); hlh-30(tm1978)	-75.74	2.50	< 0.0000000001
daf-16(mgDf47); daf-2(e1370) -> daf-16(mgDf47); daf-2(e1370); hlh-30(tm1978)	-19.63	2.06	< 0.0000000001

Figure 2c

	n	mean survival time [days]	Std.Error	independent replicate experiment (n; mean survival time [days])
glp-1(e2141)	215	16.73	0.20	131; 14.25
daf-16(mu86); glp-1(e2141)	178	10.04	0.17	329; 9.08
glp-1(e2141); hlh-30(tm1978)	214	9.31	0.07	163; 9.53
daf-16(mu86); glp-1(e2141); hlh-30(tm1978)	173	8.59	0.05	376; 8.33

	change [%]	Std.Error	log-rank test [corr. p-value]
glp-1(e2141) -> daf-16(mu86); glp-1(e2141)	-39.99	1.57	< 0.0000000001
glp-1(e2141) -> glp-1(e2141); hlh-30(tm1978)	-44.35	1.27	< 0.0000000001
glp-1(e2141) -> daf-16(mu86); glp-1(e2141); hlh-30(tm1978)	-48.66	1.23	< 0.0000000001
glp-1(e2141); hlh-30(tm1978) -> daf-16(mu86); glp-1(e2141); hlh-30(tm1978)	-7.73	0.92	< 0.0000000001

Figure 5a
oxidative stress

	n	mean survival time [hours]	Std.Error	contributing experiment #1 (n; mean survival time [days])	contributing experiment #2 (n; mean survival time [days])
wild type	147	24.96	0.45	72; 27.47	75; 22.49
daf-16(mu86)	161	14.39	0.29	82; 14.51	79; 14.29
hlh-30(tm1978)	218	14.02	0.18	108; 14.61	110; 13.46
daf-16(mu86); hlh-30(tm1978)	157	14.99	0.24	72; 14.94	85; 15.05

	change [%]	Std.Error	log-rank test [corr. p-value]
wild type -> daf-16(mu86)	-42.35	2.15	< 0.0000000001
wild type -> hlh-30(tm1978)	-43.83	1.94	< 0.0000000001
wild type -> daf-16(mu86); hlh-30(tm1978)	-39.94	2.04	< 0.0000000001
hlh-30(tm1978) -> daf-16(mu86); hlh-30(tm1978)	6.92	2.15	0.002

Figure 5b
heat stress

	n	mean survival time [days]	Std.Error	contributing experiment #1 (n; mean survival time [days])	contributing experiment #2 (n; mean survival time [days])
wild type	202 (531)	26.29 (24.93)	0.65	119; 24.43	83; 29.26
daf-16(mu86)	232 (788)	22.83 (22.97)	0.23	109; 22.63	123; 23.00
hlh-30(tm1978)	116 (677)	23.42 (23.22)	0.47	70; 22.70	46; 24.51
daf-16(mu86); hlh-30(tm1978)	273 (412)	20.36 (20.27)	0.20	118; 20.60	155; 20.17

	change [%]	Std.Error	log-rank test [corr. p-value]
wild type -> daf-16(mu86)	-15.82	3.25	< 0.0000000001
wild type -> hlh-30(tm1978)	-13.64	3.58	0.0072
wild type -> daf-16(mu86); hlh-30(tm1978)	-24.93	3.22	< 0.0000000001
hlh-30(tm1978) -> daf-16(mu86); hlh-30(tm1978)	-13.07	2.18	< 0.0000000001

Supplementary References:

1. Riedel, C. G. et al. DAF-16 employs the chromatin remodeller SWI/SNF to promote stress resistance and longevity. Nat. Cell Biol. 15, 491–501 (2013).
2. Murphy, C. T. et al. Genes that act downstream of DAF-16 to influence the lifespan of Caenorhabditis elegans. Nature 424, 277–83 (2003).
3. Massari, M. E. & Murre, C. Helix-loop-helix proteins: regulators of transcription in eucaryotic organisms. Mol. Cell. Biol. 20, 429–440 (2000).
4. Grove, C. A. et al. A multiparameter network reveals extensive divergence between C. elegans bHLH transcription factors. Cell 138, 314–27 (2009).
5. Palmieri, M. et al. Characterization of the CLEAR network reveals an integrated control of cellular clearance pathways. Hum. Mol. Genet. 20, 3852–3866 (2011).
6. Thomas-Chollier, M. et al. A complete workflow for the analysis of full-size ChIP-seq (and similar) data sets using peak-motifs. Nat. Protoc. 7, 1551–68 (2012).
